# Supplementary figures and images for: Patterns of evolution of MHC class II genes of crows (Corvus) suggest trans-species polymorphism
Source: PeerJ. 2015 Mar 19;3:e853. doi: 10.7717/peerj.853 (PMC4369332; doi:10.7717/peerj.853)

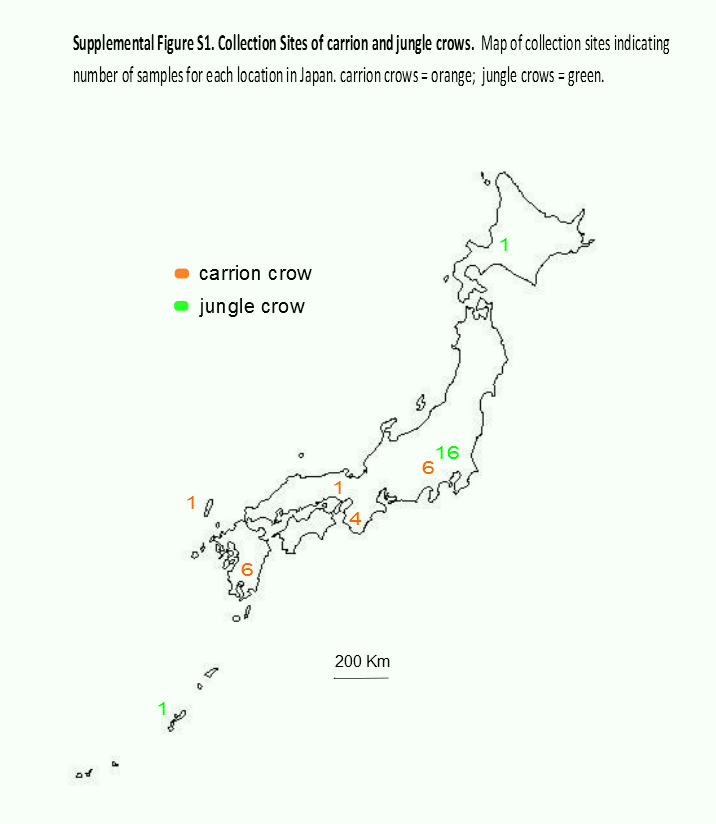

Supplement: Figure S1 — Samples numbers from each location are indicated: carrion crows, orange; jungle crows, green. [file peerj-03-853-s001.png]

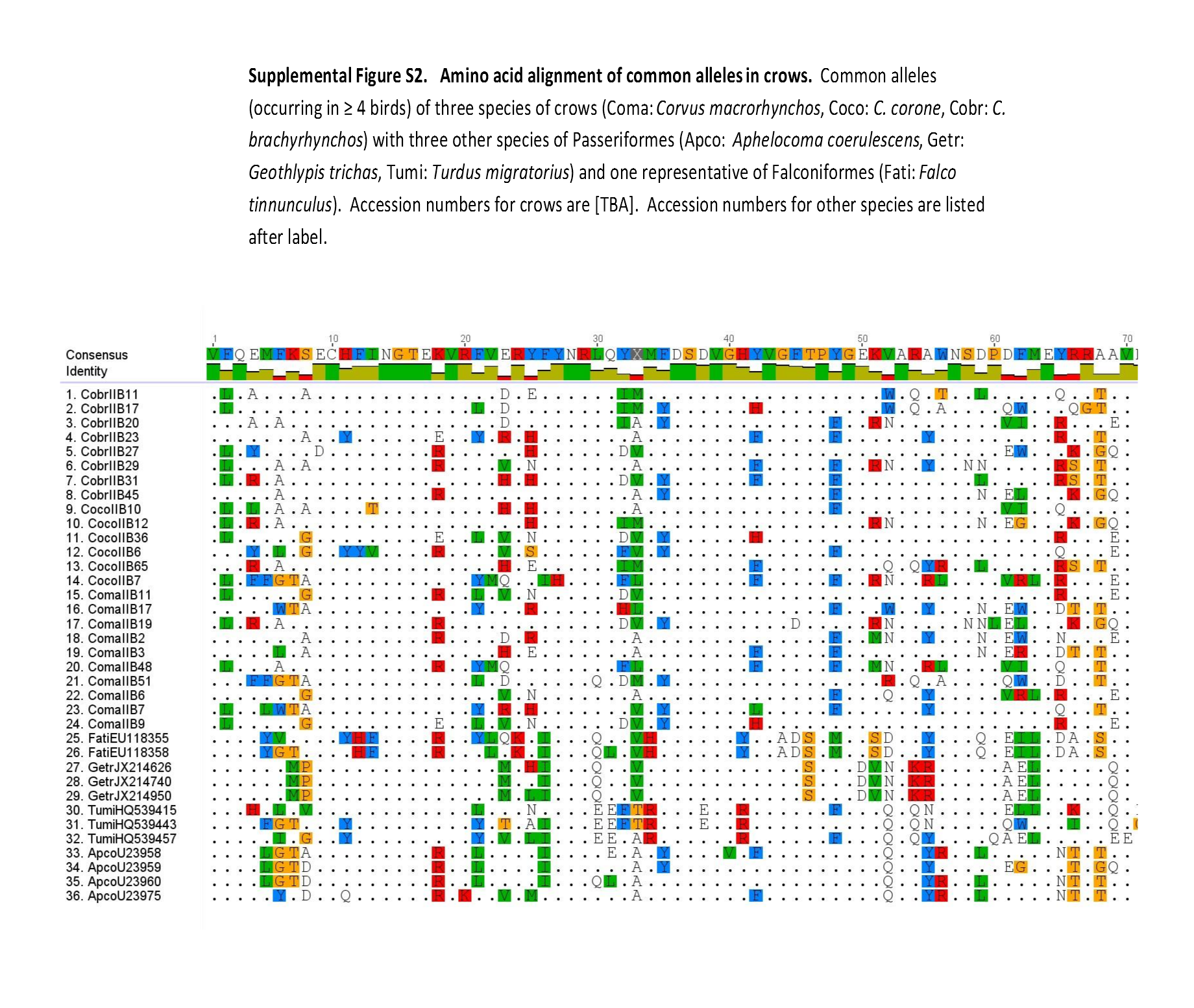

Supplement: Figure S2 — Common alleles (occurring in ≥4 birds) of three species of crows (Coma: Corvus macrorhynchos, Coco: C. corone, Cobr: C. brachyrhynchos) with three other species of Passeriformes (Apco: Aphelocoma coerulescens, Getr: Geothlypis trichas, Tumi: Turdus migratorius) and one representative of Falconiformes (Fati: Falco tinnunculus). Accession numbers for crows are [TBA]. Accession numbers for other species are listed after label. [file peerj-03-853-s002.png]

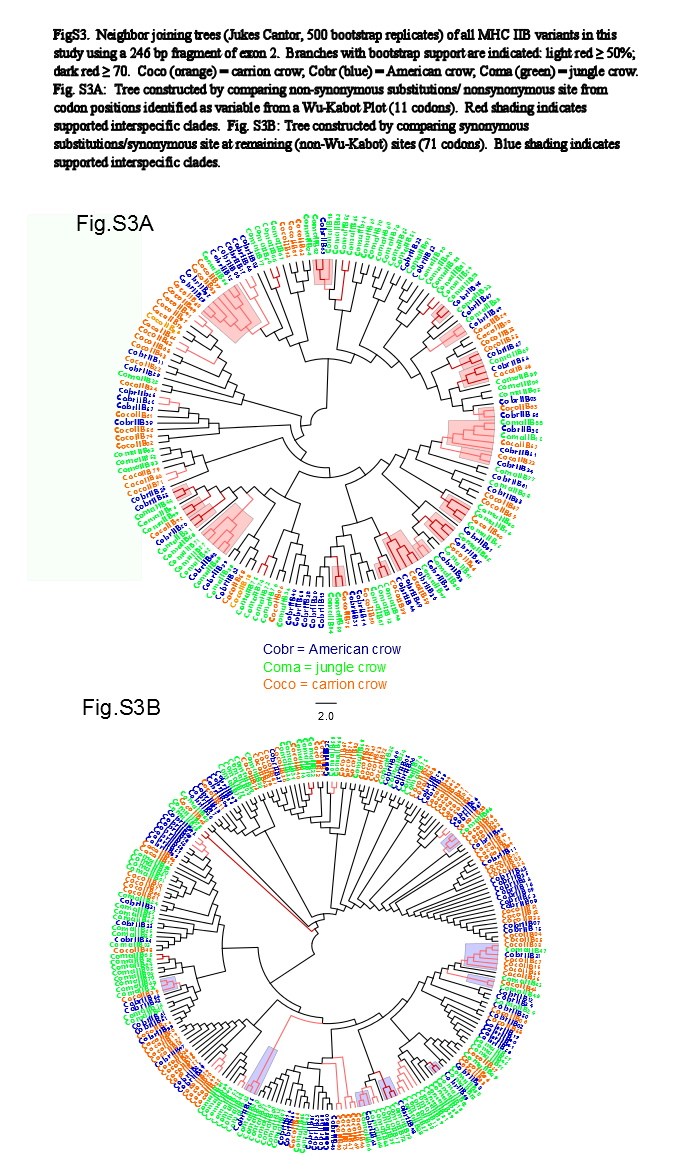

Supplement: Figure S3 — Branches with bootstrap support (Jukes-Cantor, 500 replicates) are indicated: light red ≥50%; dark red ≥70. Coco (orange), carrion crow; Cobr (blue), American crow; Coma (green), jungle crow. (S3A) Tree constructed by comparing non-synonymous substitutions/nonsynonymous site from codon positions identified as variable from a Wu-Kabot Plot (11 codons). Red shading indicates supported interspecific clades. (S3B) Tree constructed by comparing synonymous substitutions/synonymous site at remaining (non-Wu-Kabot) sites (71 codons). Blue shading indicates supported interspecific clades. [file peerj-03-853-s003.png]

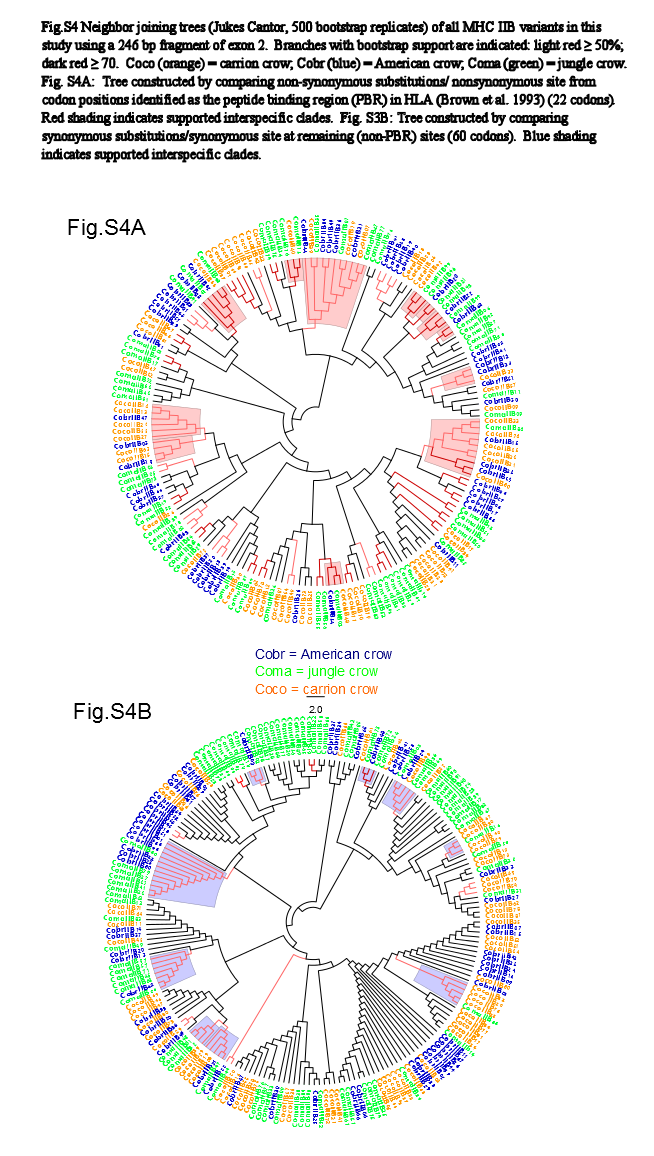

Supplement: Figure S4 — Branches with bootstrap support (Jukes-Cantor, 500 replicates) are indicated: light red ≥50%; dark red ≥70. Coco (orange), carrion crow; Cobr (blue), American crow; Coma (green), jungle crow. (S4A) Tree constructed by comparing non-synonymous substitutions/nonsynonymous site from codon positions identified as the peptide binding region (PBR) in HLA (Brown et al., 1993) (22 codons). Red shading indicates supported interspecific clades. (S3B) Tree constructed by comparing synonymous substitutions/synonymous site at remaining (non-PBR) sites (60 codons). Blue shading indicates supported interspecific clades. [file peerj-03-853-s004.png]
