# Supplementary material for: Patterns of evolution of MHC class II genes of crows (Corvus) suggest trans-species polymorphism
Source: PeerJ. 2015 Mar 19;3:e853. doi: 10.7717/peerj.853 (PMC4369332; doi:10.7717/peerj.853)
Supplement: Table S1 — Primers ComaIIbex2r and ComaEx2RA were ligated to fusion primers for 454 sequencing. [file peerj-03-853-s005.docx]

**Supplemental Table S1.** Primers used in this study to amplify cDNA and gDNA. Primers ComaIIbex2r and ComaEx2RA were ligated to fusion primers and MIDs for 454 sequencing.

| Name | Sequence | Annealing Temp |
| --- | --- | --- |
| MHC05 | CGTRCTGGTGGCACTGGTGGYGCT | 55 |
| ComaIIbex3R | CAGYGAGATGGACACGCTGGG | 55 |
| ComaIIbex2R | GTGGACACCTCGTAGTTGT | 57 |
| ComaiF2 | CCTGTGTCCCGCACAAACAGGG | 57 |
| ComaEx2RA | CTCGGCGCTCCACKSTGAAC | 57 |
| CR-COR+ | ACCCTTCAAGTGCGTAGCAG | 60 |
| Phe-Cor- | TGACATCTTCAGTGTCATGC | 60 |
